# Determination of *in situ* ruminal degradation of phytate phosphorus from single and compound feeds in dairy cows using chemical analysis and near-infrared spectroscopy

**DOI:** 10.1017/S1751731120000221

**Published:** 2020-07

**Authors:** E. Haese, J. Krieg, G. Grubješić, A. Feyder, M. Rodehutscord

**Affiliations:** Institut für Nutztierwissenschaften, Universität Hohenheim, Emil-Wolff-Str. 6-10, 70599 Stuttgart, Germany

**Keywords:** feed evaluation, phosphorus availability, phytate degradation, rumen, analytical method

## Abstract

The ruminal degradation of P bound in phytate (**InsP**_**6**_) can vary between feeds, but data on ruminal degradation of InsP_6_ from different feedstuffs for cattle are rare. One objective of this study was to increase the data base on ruminal effective degradation of InsP_6_ (**InsP**_**6**_ **ED**) and to assess if InsP_6_ED of compound feeds (**CF**) can be calculated from comprising single feeds. As a second objective, use of near-infrared spectroscopy (**NIRS**) to predict InsP_6_ concentrations was tested. Nine single feeds (maize, wheat, barley, faba beans, soybeans, soybean meal (**SBM**), rapeseed meal (**RSM**), sunflower meal (**SFM**), dried distillers’ grains with solubles (**DDGS**)) and two CF (**CF1**/**CF2**), consisting of different amounts of the examined single feeds, were incubated for 2, 4, 8, 16, 24, 48 and 72 h in the rumen of three ruminally fistulated Jersey cows. Samples of CF were examined before (**CF1**/**CF2 Mash**) and after pelleting (**CF1/CF2 Pellet**), and InsP_6_ED was calculated for all feeds at two passage rates (**InsP**_**6**_ **ED**_**5**_: *k* = 5%/h; **InsP**_**6**_ **ED**_**8**_: *k* = 8%/h). For CF1 and CF2, InsP_6_ED was also calculated from values of the respective single feeds. Near-infrared spectra were recorded in duplicate and used to establish calibrations to predict InsP_6_ concentration. Besides a global calibration, also local calibrations were evaluated by separating samples into different data sets based on their origin. The InsP_6_ED_8_ was highest for faba beans (91%), followed by maize (90%), DDGS (89%), soybeans (85%), wheat (76%) and barley (74%). Lower values were determined for oilseed meals (48% RSM, 65% SFM, 66% SBM). Calculating InsP_6_ED of CF from values of single feeds underestimated observed values up to 11 percentage points. The NIRS calibrations in general showed a good performance, but statistical key data suggest that local calibrations should be established. The wide variation of InsP_6_ED between feeds indicates that the ruminal availability of P bound in InsP_6_ should be evaluated individually for feeds. This requires further *in situ* studies with high amounts of samples for InsP_6_ analysis. Near-infrared spectroscopy has the potential to simplify the analytical step of InsP_6_ in the future, but the calibrations need to be expanded.

## Implications

Phosphorus is essential for health, milk production and reproduction of dairy cows but contributes to environmental pollution when excreted. In plant seeds, P is mainly stored as phytate, but phytate degradation and, thus, availability of P in the rumen vary widely between different feeds. Data on ruminal phytate degradation of feeds commonly fed to dairy cows improves diet calculations contributing to an adequate P supply of the animals. In the future, the data base on ruminal phytate degradation can be further increased when near-infrared spectroscopy is used to predict phytate concentrations instead of elaborate chemical analysis.

## Introduction

An adequate supply of P is essential to ensure health and performance of dairy cows. However, faecal P excretion increases with P intake in a linear manner (Wu *et al.*, 2001), and P concentrations in the diet exceeding the animals’ requirement lead to increased faecal P excretion. Phosphorus losses can contribute to eutrophication of natural waters (Desmit *et al.*, 2018) and, thus, excessive P supply in animal nutrition has to be avoided.

In plant seeds and by-products, P is contained predominantly as phytate (any salt of phytic acid; *myo*-inositol 1,2,3,4,5,6-hexakis (dihydrogen phosphate); **InsP**_**6**_). Rumen microorganisms show substantial phytase activity (Yanke *et al.*, 1998) which enables the hydrolytic cleavage of P bound in InsP_6_ (**InsP**_**6**_ **-P**) and subsequent P absorption in the intestine. However, results of studies examining total tract disappearance of InsP_6_ are inconsistent. While several studies found only low faecal InsP_6_ excretion of about 5% of ingested InsP_6_ (e.g. Morse *et al.*, 1992; Ray *et al.*, 2013), others reported higher proportions of InsP_6_ excreted (e.g. Haese *et al.*, 2014: up to 15%; Kincaid *et al.*, 2005: more than 20% of ingested InsP_6_). Some of the observed differences can likely be explained by the wide variation of feed ingredients used in the diets. Earlier *in vitro* and *in situ* studies have shown that progression and extent of ruminal InsP_6_ disappearance differ between feedstuffs. In rapeseed meal (**RSM**), InsP_6_ disappearance proceeded slowly compared to maize (Haese *et al.*, 2017a), soybean meal (**SBM**) and wheat (Haese *et al.*, 2017b), leading to a lower effective InsP_6_ degradation of RSM in the rumen compared to SBM (Park *et al.*, 1999). However, data on effective degradation of InsP_6_ (**InsP**_**6**_ **ED**) in common feeds for cattle are rare to date. Thus, the first objective of the present study was to determine InsP_6_ED from different single feeds used in cattle feeding. Furthermore, we determined InsP_6_ED of compound feeds (**CF**) to assess if InsP_6_ degradation values from single feeds are additive in CF. This would allow for calculations of InsP_6_ED for any compound feed if respective values are given for the utilised single feeds. Increased data on the ruminal availability of InsP_6_-P from different feeds may allow for more precise calculation of dietary P supply of dairy cows in the future.

*In situ* studies to determine InsP_6_ED provide a large number of samples to be analysed for inositol phosphates (**InsPs**). Most commonly, high-performance ion chromatography (**HPIC**) with gradient elution or similar chromatography is used to separate InsPs and their isomers in feeds (Blaabjerg *et al.*, 2010). However, this technique is laborious and costly and is not established as a routine method for common feed analysis. Hence, faster and easier methods for analysis of InsP_6_ would be beneficial to increase the data base of ruminal InsP_6_ degradation of feeds. Various studies showed that near-infrared spectroscopy (**NIRS**) can be used to predict the concentration of InsP_6_ (Zhao *et al.*, 2017) and InsP_6_-P (Tahir *et al.*, 2012; Aureli *et al.*, 2017), while studies that applied this technique to *in situ* samples were not reported. However, for cereal grains, NIRS has been successfully used to predict CP and starch in bag residues after ruminal incubation (Krieg *et al.*, 2018a). Hence, the second objective of this study was to establish calibrations to predict the InsP_6_ concentration of feeds and ruminally incubated bag residues using NIRS. In order to examine the suitability of NIRS estimations for the usage in *in situ* studies, InsP_6_ED calculated from NIRS-derived InsP_6_ concentrations was compared to those calculated from chemically analysed InsP_6_ concentrations of the samples.

## Material and methods

### Samples and incubations

Samples of single and compound feeds and their respective bag residues originated from an *in situ* study described in detail by Grubješić *et al.* (2019). Nine single feeds (maize, wheat, barley, faba beans, soybeans, SBM, RSM, sunflower meal (**SFM**), dried distillers’ grains with solubles (**DDGS**)) and two CF (**CF1, CF2**) composed of different amounts of these single feeds were used for analysis of InsPs. Compound feed 1 consisted of 10% maize, 46% barley, 16% faba beans, 18% soybeans, 5% SBM and 5% DDGS, while CF2 contained 32% maize, 12% wheat, 16% faba beans, 8% SBM, 17% RSM, 10% SFM and 5% DDGS (values on DM basis). The CF were produced in a commercial feed mill as described in detail by Grubješić *et al.* (2019). In brief, single feeds were ground through a 3 mm sieve and mixed into the CF. Subsequently, one portion of the compound feed was pelleted at 50°C to 60°C (exit temperature 80°C to 90°C). For the *in situ* incubations of CF1 and CF2, samples were taken before (**Mash**) and after pelleting (**Pellet**).

The ruminal incubation followed the procedure of Seifried *et al.* (2017) and was also described in detail by Grubješić *et al.* (2019). In brief, feed samples were ground to pass a 2 mm sieve and 8 g were weighed into polyester bags (10 × 20 cm, pore size 50 μm, ANKOM Technology, USA) with 3 to 5 replicates per sample, incubation time and animal. The bags were incubated in the rumen of three rumen-fistulated Jersey cows for 2, 4, 8, 16, 24, 48 and 72 h and washed in a washing machine after incubation. Values for incubation time 0 h were gained by washing three replicates of each feed sample in the washing machine without ruminal incubation. For analysis, the dried replicates were weighed and pooled per feed sample, incubation time and animal.

### Chemical analysis

Dry matter of feed samples and bag residues was analysed according to the official methods used in Germany (Verband Deutscher Landwirtschaftlicher Untersuchungs- und Forschungsanstalten, 2007). Analysis of InsP_6_ and isomers of lower InsPs (myo-inositol pentakisphosphate (**InsP**_**5**_), myo-inositol tetrakisphosphate (**InsP**_**4**_) and myo-inositol trisphosphate (**InsP**_**3**_)) was performed as described by Zeller et al. (2015) with slight modifications regarding sample size and agent used for extraction. In brief, 0.1 g of the sample was extracted for 30 min with 1.0 ml of an extracting agent (0.2 Mol ethylenediaminetetraacetate and 0.1 Mol NaF, pH 8.0) on a rotary shaker. After centrifugation, the supernatant was removed, preserved on ice and the residue re-suspended with 0.5 ml extracting agent and extracted again for 30 min. The supernatants of both extraction steps were merged, filtered and centrifuged. Filtrates were analysed by HPIC (ICS-3000, Fa. Dionex, Idstein, Germany) and UV detection at 290 nm.

### Calculations

For each feed, degradation parameters ***a*** (%; rapidly disappearing fraction), ***b*** (%; potentially degradable fraction), ***a + b*** (%; maximum degradation/plateau) and ***c*** (%/h; degradation rate) of InsP_6_ were calculated based on HPIC-derived InsP_6_ concentrations using the equations described by Orskov and McDonald (1979) (equation (1)) and McDonald (1981) (equation (2)).(1)
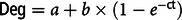

(2)
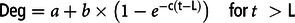
where Deg (%) is the ruminal degradation of InsP_6_ after *t* h and L represents lag time. Using the GraphPad Prism software (Version 5.0 for Windows, GraphPad Software, CA, USA), the best fitting model for each feed was selected based on the Akaike Information Criterion. For estimation of degradation values, estimations of fraction *a* and fraction *a* + *b* were constrained to 0 and 100%, respectively. The degradation parameters of InsP_6_ were then used to calculate the InsP_6_ED at ruminal outflow rates of *k* = 5 (**InsP**_**6**_ **ED**_**5**_) or 8 (**InsP**_**6**_ **ED**_**8**_) %/h with either(3)
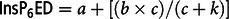



according to Orskov and McDonald (1979) or(4)
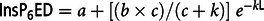



according to Wulf and Südekum (2005).

For the CF, the degradation parameters and InsP_6_ED values were additionally calculated from the observed values of single feeds as described by Grubješić *et al.* (2019) using(5)
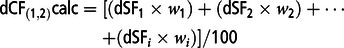

dCF_(1,2)_calc = calculated degradation characteristics (*a*, *b*, *c*, lag, InsP_6_ED_5_, InsP_6_ED_8_) of CF1 or CF2
dSF_*i*_ = observed degradation characteristics (*a*, *b*, *c*, lag, InsP_6_ED_5_, InsP_6_ED_8_) of single feed *i*
*w*_*i*_ = weighted InsP_6_ contribution of single feed *i* to total InsP_6_ pool of CF1 or CF2

Degradation parameters and InsP_6_ED were calculated for each cow separately, using cow as experimental unit in statistical analysis.

### Near-infrared spectroscopy

Because the number of feeds used in this study was relatively low for developing NIRS calibrations for InsP_6_, values of samples from earlier *in situ* studies were added to the data pool. All additional data originated from studies where different feeds were ruminally incubated and analysed for InsP_6_ concentrations using HPIC as described before. The additional data included values for barley, maize, rye, triticale and wheat (Seifried *et al.*, 2016 and 2017; Krieg *et al.*, 2017) and four RSM samples (Haese *et al.*, 2017c). Different combinations of samples were tested for the establishment of calibrations in order to compare the performance of local calibrations (including only one type of feed, e.g. cereal grains) with global calibrations (including all feed types) and to achieve the overall best performance. A total of seven data sets was created using different combinations of feeds and corresponding bag residues:

Data set 1: all values for feeds and bag residues of the present study
Data set 2: all values for feeds and bag residues of the present study and the additional studies (Seifried *et al.*, 2016 and 2017; Haese *et al.*, 2017c; Krieg *et al.*, 2017)
Data set 3: data set 2, but excluding all values for rye and triticale
Data set 4: only values for feeds and bag residues from grain samples of the present study and the additional studies
Data set 5: data set 2, but excluding all values for grain samples
Data set 6: data set 2, but excluding all values for CF
Data set 7: data set 2, but excluding all values for CF and grain samples.

Number of samples used for calibration and validation data sets are shown in Table 1.

**Table 1:** Number (*n*) of feed samples used for calibration development and validation. Mean and range of chemically analysed phytate (InsP_6_) concentration of feeds and bag residues after *in situ* incubation`

	Calibration				Validation			
	*n*	Mean	Min	Max	*n*	Mean	Min	Max
		(µmol/g DM)				(µmol/g DM)		
All^1^	259	18.1	1.3	66.5	102	18.4	1.3	65.2
Maize^1,2^	24	8.8	1.3	16.6	10	8.7	1.6	15.4
Wheat^1,3^	24	15.0	1.9	43.9	9	16.1	2.9	41.0
Barley^1,4^	25	14.6	2.1	28.8	9	14.2	2.8	21.4
Faba beans^1^	10	10.0	2.1	21.7	4	9.2	1.3	16.6
Soybeans^1^	10	13.6	2.8	21.9	4	13.6	3.7	18.6
Soybean meal^1^	9	10.7	31.6	25.0	4	10.9	31.4	23.7
Rapeseed meal^1,5^	69	31.0	1.3	66.5	27	30.2	1.6	65.2
Sunflower meal^1^	9	7.1	63.5	39.3	5	7.1	63.3	42.8
DDGS^1^	10	3.3	1.5	7.0	4	3.0	1.6	1.6
CF1, CF2 Mash^1^	20	13.9	2.1	25.3	8	13.7	2.3	25.0
CF1, CF2 Pellet^1^	19	11.5	2.2	20.4	8	10.9	2.3	20.3
Rye^4^	15	8.0	6.6	9.8	5	8.1	6.7	9.2
Triticale^4^	15	10.1	8.5	13.6	5	9.9	8.6	11.0

Min = minimum value; Max = maximum value.

Samples of ^1^the present study, ^2^Seifried *et al.* (2016) ^3^Seifried *et al.* (2017) ^4^ Krieg *et al.* (2017), ^5^Haese *et al.* (2017c).

DDGS = dried distillers’ grains with solubles; CF1 = compound feed 1 (containing 10% maize, 46% barley, 16% faba beans, 18% soybeans, 5% soybean meal, 5% DDGS on DM basis); CF2 = compound feed 2 (containing 32% maize, 12% wheat, 16% faba beans, 8% soybean meal, 17% rapeseed meal, 10% sunflower meal, 5% DDGS on DM basis).

Spectra were recorded in duplicate from 680 to 2500 nm (SpectraStar 2500X, Software: Unity InfoStar Version 3.11.1, Unity Scientific, Brookfield, CT). Additionally, spectra of an internal standard as well as external standards (US-STDS-0001 – STD, Wavelength cert, R99 and US-STDS-0003 – STD, Wavelength cert, R99/Poly; Unity Scientific, Brookfield, CT) were recorded throughout the measurements. Mathematical treatment of the spectra and calibrations computation were carried out using the software Ucalibrate (Version: 3.0.0.23; Unity Scientific, Brookfield, CT). The spectra were averaged per sample, and the averaged spectrum of each sample was mathematically pre-treated by standard normal variates and detrending. Derivations of the spectra were computed using a derivation gap and smoothing steps of eight. The derivation option varied between no derivation and first- or second-order derivation. Subsequently, the spectra were used for calibration calculation. The samples were split into a calibration and a validation set for each feed type as outlined in Table 1, attempting to include the whole range of InsP_6_ concentrations in both calibration and validation sets.

Three wavelength segments were compared: (1) the complete recorded spectrum (680–2500 nm), (2) the recorded spectrum constricted for 50 nm from the beginning and the end (730 to 2450 nm) and (3) the segment of 1250 to 2450 nm. Segment 2 was used to eliminate possible drifts near the limit of the detection. Segment 3 was used because most N–H and C–H bonds are known to be located in this area and because the protein and InsP_6_ concentration correlated in RSM and SBM after ruminal *in situ* incubation (Haese *et al.*, 2017b). Each of the three wavelength segments was combined with each derivation, resulting in nine calibrations per data set. Stepwise forward partial least squares (PLS)-regression was used to compute calibrations. Number of groups for cross validation (**CV**) varied, depending on the number of samples in the calibrations. The T-limit for outlier detection was set to 2.5 (predicted *v*. reference value), and global distance limit was set to 13.

Calibration evaluation was carried out using the standard error of calibration (**SEC**) and the standard error of prediction (**SEP**) as a measure for the accuracy of the calibration (Bellon-Maurel *et al.,* 2010). Coefficients of determination (predicted *v*. reference) were also considered. The performance of the calibrations was further evaluated using the bias, the intercept and the slope of the validation step. The target values were zero for the bias and the intercept and one for the slope.

To evaluate the suitability of NIRS as alternative method to HPIC in *in situ* experiments, InsP_6_ED was additionally calculated based on NIRS predicted InsP_6_ concentrations of the feeds and bag residues according to equations 1 to 4 (**InsP**_**6**_ **ED NIRS**). The InsP_6_ concentrations were predicted using the most accurate calibration and data set. These InsP_6_ED values were then compared to InsP_6_ED values deduced from InsP_6_ concentrations measured using HPIC (**InsP**_**6**_ **ED HPIC**).

### Statistical analysis

Degradation parameters *a*, *b*, *c* and lag as well as InsP_6_ED values were statistically analysed with the SAS MIXED procedure (SAS System for Windows, Version 9.4, SAS Institute, Cary, NC, USA). For single feeds, a one-factorial approach with the following model was used:
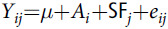
with *Y*_*ij*_ as responsive mean, μ as overall mean, *A*_*i*_ as random effect of animal (*i* = 1, 2, 3), SF_j_ as fixed effect of single feed (*j* = maize, wheat, barley, faba beans, soybeans, SBM, RSM, SFM, DDGS) and *e*_*ij*_ as residual error.

Compound feeds were analysed in a two-factorial approach with the model:
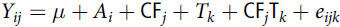
where CF_*j*_ is the fixed effect of compound feed (*j* = CF1, CF2), *T*_*k*_ is the fixed effect of type (*k* = Mash, Pellet, Calculated), and CF_*j*_ *T*_*k*_ is the interaction of CF_*j*_ and *T*_*k*_. Data are presented as least-squares means (LS means) and pooled standard error of the means (pooled SEM).

For comparison of the InsP_6_ED values based on chemical and NIRS derived InsP_6_ concentrations also a two-factorial approach was used:
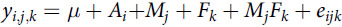
where *M*_*j*_ is the method used to determine InsP_6_ concentration (*j* = HPIC, NIRS), *F*_*k*_ the feed (*k* = maize, wheat, barley, faba beans, soybeans, SBM, RSM, SFM, DDGS, CF1 Mash, CF2 Mash, CF1 Pellet, CF2 Pellet), and M_j_F_k_ is the interaction of M_j_ and F_k_.

Statistical significance was declared at *P* < 0.05 for all models. Following a significant *F* value, *t*-tests were performed to show individual significant differences between means.

## Results

### Concentrations of inositol phosphates in single and compound feeds

The concentration of InsP_6_ varied from 7.0 µmol/g DM (4.6 g/kg DM) to 49.9 µmol/g DM (32.9 g/kg DM) between the examined feeds (Table 2), with the lowest InsP_6_ concentrations in DDGS and cereal grains (7.0 to 12.4 µmol/g DM; 4.6 to 8.2 g/kg DM) and the highest in RSM and SFM (36.5 and 49.9 µmol/g DM; 24.1 and 32.9 g/kg DM, respectively). The InsP_6_ concentrations in CF1 (Mash and Pellet) were considerably lower compared to CF2.

**Table 2:** Concentrations of phytate (InsP_6_) and myo-inositol pentakisphosphate (InsP_5_) in the examined single and compound feeds^1^(µmol/g DM and g/kg DM)

		InsP_6_		InsP_5_	
Feed		µmol/g DM	g/kg DM	µmol/g DM	g/kg DM
Maize		10.7	7.0	0.3*	0.2*
Wheat		12.4	8.2	0.3*	0.2*
Barley		9.6	6.3	0.3*	0.2*
Faba beans		21.7	14.3	2.7	1.6
Soybeans		21.8	14.4	3.9	2.2
Soybean meal		25.8	17.0	3.8	2.2
Rapeseed meal		36.5	24.1	5.4	3.2
Sunflower meal		49.9	32.9	7.5	4.4
DDGS		7.0	4.6	3.9	2.2
CF1	Mash	13.2	8.7	2.0	1.2
	Pellet	13.5	8.9	1.5	0.9
CF2	Mash	21.8	14.4	2.9	1.7
	Pellet	19.1	12.6	2.5	1.5

DDGS = dried distillers’ grains with solubles; CF1 = compound feed 1 (containing 10% maize, 46% barley, 16% faba beans, 18% soybeans, 5% soybean meal, 5% DDGS on DM basis); CF2 = compound feed 2 (containing 32% maize, 12% wheat, 16% faba beans, 8% soybean meal, 17% rapeseed meal, 10% sunflower meal, 5% DDGS on DM basis).

*Below limit of quantification, approximate value (mean between limit of detection and limit of quantification).

1 Chemical composition of the feeds besides inositol phosphates published by Grubješić *et al.* (2019).

In cereal grains, only traces of InsP_5_ were determined (below limit of quantification, approximately 0,3 µmol/g DM). In the other feeds, InsP_5_ concentrations ranged from 1.5 µmol/g DM to 7.5 µmol/g DM (Table 2). The highest InsP_5_ concentrations were determined in RSM and SFM (5.4 and 7.5 µmol/g DM, respectively). Concentrations of InsPs lower than InsP_5_ overall were very low and only for DDGS slightly above the quantification limit (1.4 µmol/g DM InsP_4_ and 1.5 µmol/g DM InsP_3_, data not shown).

### Degradation parameters and effective degradation of phytate from single feeds

Ruminal degradation parameters *a*, *b* and *c* differed significantly between the single feeds and ranged from 0% (RSM) to 77% (DDGS) for fraction *a*, from 22% (DDGS) to 100% (RSM) for fraction *b* and from 7.3%/h (RSM) to 28.2%/h (SFM) for degradation rate *c* (Table 3). The InsP_6_ED also varied widely between feeds for both calculated passage rates and was highest for faba beans, maize and DDGS (InsP_6_ED_5_: 93, 93 and 92%; InsP_6_ED_8_: 91, 90 and 89%, respectively), followed by soybeans, wheat and barley (InsP_6_ED_5_: 89, 82, 80%; InsP_6_ED_8_: 85, 76, 74%, respectively; Table 3). In the oilseed meals, InsP_6_ED was lowest with values for InsP_6_ED_5_ and InsP_6_ED_8_ of 76 and 66% for SBM, 75 and 65% for SFM and 59 and 48% for RSM, respectively. A significant lag time was only calculated for SBM (3.6 h) and SFM (3.1 h).

**Table 3:** Ruminal degradation parameters and effective degradation of phytate (InsP_6_) for single feeds (*n* = 3 animals)

	Maize	Wheat	Barley	Faba beans	Soybeans	Soybean meal	Rapeseed meal	Sunflower meal	DDGS	Pooled SEM	*P*-values
*a*	63^c^	45^ d^	44^ d^	74^b^	62^c^	27^e^	0^ g^	15^f^	77^a^	0.66	<0.001
*b*	37^e^	55^ d^	56^ d^	26^f^	38^e^	73^c^	100^a^	84^b^	22^ g^	0.71	<0.001
*c*	24.9^ab^	10.2^ d^	9.4^ d^	14.9^bcd^	12.2^ cd^	20.7^abc^	7.3^d^	28.2^a^	10.8^cd^	3.48	0.005
lag	–	–	–	–	–	3.6^a^	–	3.1^b^	–	0.09	0.005
InsP_6_ED_5_	93^a^	82^c^	80^c^	93^a^	89^b^	76^ d^	59^e^	75^ d^	92^a^	0.86	<0.001
InsP_6_ED_8_	90^a^	76^c^	74^c^	91^a^	85^b^	66^ d^	48^e^	65^ d^	89^a^	1.11	<0.001

*a* = rapidly degradable fraction (%); *b* = potentially degradable fraction (%); *c* = degradation rate of *b* (%/h); lag = lag time (h); InsP_6_ED = effective degradation (%) of InsP_6_ at a passage rate of 5 (InsP_6_ED_5_) and 8 (InsP_6_ED_8_) %/h.

DDGS = dried distillers’ grains with soluble.

Different superscripts within a row indicate significant differences.

### Degradation parameters and effective degradation of phytate from compound feeds

In CF, fraction *a* was significantly higher for both CF Pellets compared to their respective Mash (CF1: 71 *v*. 56%, CF2: 56 *v*. 38%; Table 4). The same was observed for InsP_6_ED_5_ (CF1: 91 *v*. 86%, CF2: 85 *v* 80%) and InsP_6_ED_8_ (CF1: 88 *v*. 81%, CF2: 80 *v*. 72%). For fraction *c*, no interactions between feed and type existed, but the degradation rate was significantly higher for CF2 compared to CF1 (17.5 *v*. 11.2%/h). Calculated values for fraction *a*, InsP_6_ED_5_ and InsP_6_ED_8_ did not differ from observed values for CF1 Mash but were lower than the observed values of CF1 Pellet. For CF2, calculated values for fraction *a*, InsP_6_ED_5_ and InsP_6_ED_8_ were lower than the observed values of CF2 Mash and CF2 Pellet.

**Table 4:** Ruminal degradation parameters and effective degradation of phytate (InsP_6_) for compound feeds (CF1/2 Mash, CF1/2 Pellet and CF1/2 Calculated, *n* = 3 animals)

	CF1			CF2			Pooled SEM	CF1	CF2	Pooled SEM	*P*-values		
Type	Mash	Pellet	Calculated	Mash	Pellet	Calculated					CF × Type	CF	Type
*a*	56^b^	71^a^	57^b^	38^c^	56^b^	32^ d^	0.95				<0.001	<0.001	<0.001
*b*	44^c^	29^ d^	43^c^	61^b^	43^c^	68^a^	0.91				<0.001	<0.001	<0.001
*c*	10.5	11.1	12.0	18.0	20.1	14.4	–	11.2	17.5	1.65	0.442	0.014	0.662
lag	–	–	0.3^ d^	2.5^b^	3.5^a^	1.0^c^	0.18				<0.001	<0.001	<0.001
InsP_6_ED_5_	86^b^	91^a^	87^b^	80^c^	85^b^	77^ d^	0.73				0.022	<0.001	<0.001
InsP_6_ED_8_	81^b^	88^a^	82^b^	72^c^	80^b^	69^ d^	0.87				0.030	<0.001	<0.001

*a* = rapidly degradable fraction (%); *b* = potentially degradable fraction (%); *c* = degradation rate of *b* (%/h); lag = lag time (h); InsP_6_ED = effective degradation (%) of InsP_6_ at a passage rate of 5 (InsP_6_ED_5_) and 8 (InsP_6_ED_8_) %/h.

CF1 = compound feed 1 (containing 10% maize, 46% barley, 16% faba beans, 18% soybeans, 5% soybean meal, 5% dried distillers’ grains with solubles (DDGS) on DM basis); CF2 = compound feed 2 (containing 32% maize, 12% wheat, 16% faba beans, 8% soybean meal, 17% rapeseed meal, 10% sunflower meal, 5% DDGS on DM basis).

CF Calculated = ruminal degradation parameters and effective degradation of InsP_6_ calculated from single feeds.

Different superscripts within a row indicate significant differences.

### Concentrations of lower inositol phosphates after different incubation times

**Figure 1:**
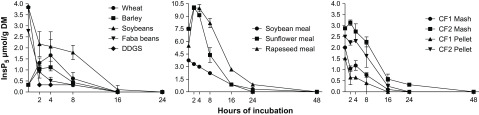
Concentrations of *myo*-inositol pentakisphosphate (InsP_5_; μmol/g DM) in the bag residues of *in situ* incubated single and compound feeds at different incubation times (*n* = 3 animals; DDGS = dried distillers’ grains with solubles; CF1 = compound feed 1 (containing 10% maize, 46% barley, 16% faba beans, 18% soybeans, 5% soybean meal, 5% DDGS on DM basis); CF2 = compound feed 2 (containing 32% maize, 12% wheat, 16% faba beans, 8% soybean meal, 17% rapeseed meal, 10% sunflower meal, 5% DDGS on DM basis).

Isomers of InsP_5_ were detected in the bag residues of all incubated feeds except for maize. Concentrations of InsP_5_ in the bag residues during the course of incubation are shown in Figure 1. Compared to the concentrations in the feeds, the InsP_5_ concentrations in the bag residues initially increased for wheat, barley, RSM, SFM and CF2 Mash after 2 or 4 h but decreased quickly afterwards. Only traces of InsP_5_ were detected in the bag residues after 16 h (wheat, barley, soybeans, faba beans, DDGS) or 24 h of incubation (SBM, RSM, SFM, CF1, CF2). Inositol phosphates lower than InsP_5_ were only found in the form of InsP_4_ in the bag residues of SFM (after 2 and 4 h) and RSM (after 4 h of incubation), but the concentrations were negligible (data not shown).

### Near-infrared spectroscopy calibrations

**Figure 2:**
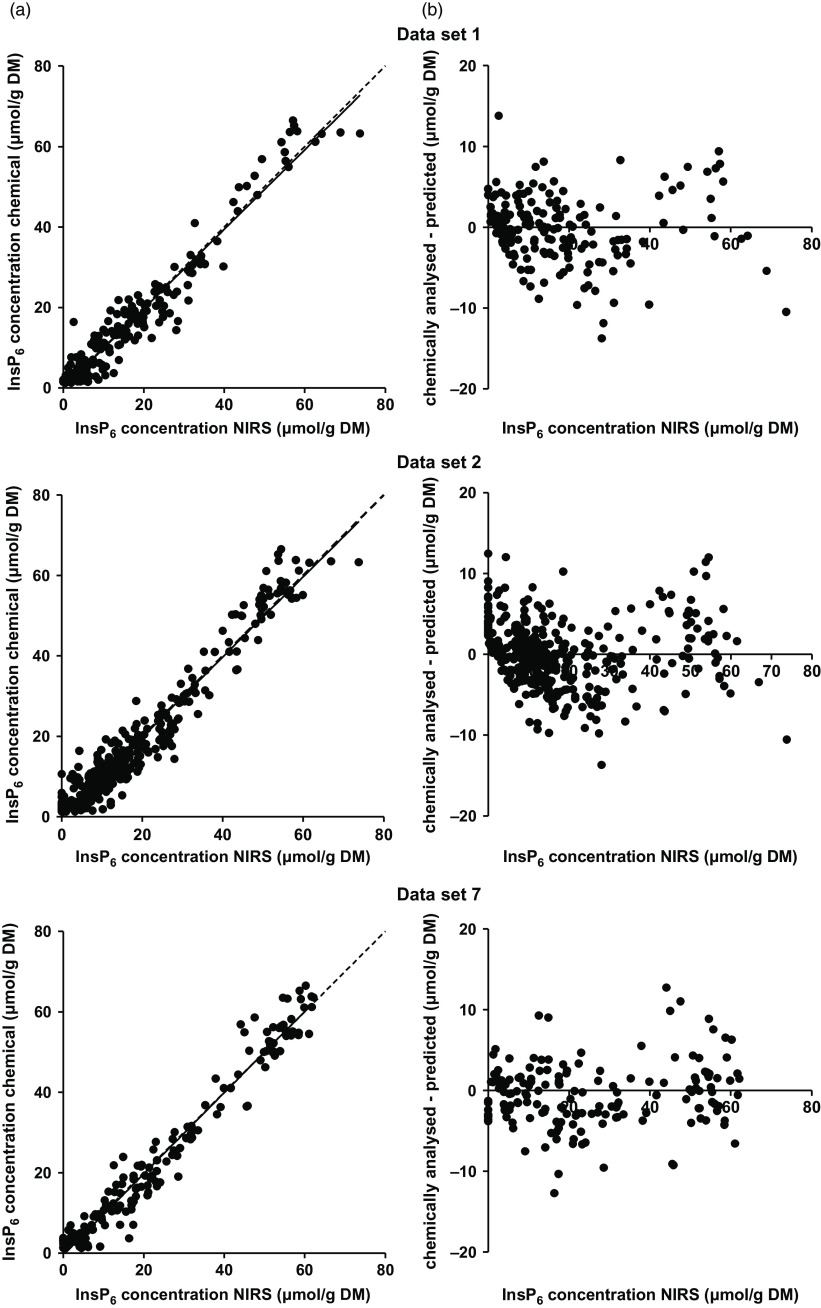
(a) Phytate (InsP_6_) concentrations (predicted with near-infrared spectroscopy (NIRS) vs. chemically analysed) in samples from *in situ* studies based on data sets 1, 2 and 7, the corresponding regression line (solid line) and the bisectrix (dashed line). (b) Difference between NIRS predicted and chemically analysed InsP_6_ concentrations in samples of *in situ* studies. Negative values were treated as zero.

The calibration based on data set 7 showed the highest *R* ^2^ values and the lowest error measurements (Table 5, Figure 2). For all data sets, the first derivation of the spectra showed the best performance. With the exception of data set 4, the calibration based on the wavelength segment of 1250 to 2450 nm was chosen for all data sets as the best performing one. Deviation of the prediction from the chemically determined InsP_6_ concentration against the predicted value was homogeneously distributed across the whole range of predictions (Figure 2). The InsP_6_ concentrations of feeds and bag residues derived from data set 7 were then used to calculate InsP_6_ED NIRS for comparison with InsP_6_ED HPIC (Table 6). Significant differences in InsP_6_ED values occurred for some feeds. For wheat, barley and CF1 Mash, InsP_6_ED_8_ NIRS was up to 10 percentage points higher compared to InsP_6_ED_8_ HPIC. On the other hand, InsP_6_ED_8_ NIRS for maize, SBM and SFM was up to 16 percentage points lower compared to InsP_6_ED_8_ HPIC. For the other feeds (faba beans, soybeans, RSM, DDGS, CF1 Pellet, CF2 Mash and CF2 Pellet), InsP_6_ED NIRS and InsP_6_ED HPIC did not differ significantly.

**Table 5:** Performance of different calibrations for estimating the phytate (InsP_6_) concentration of single feeds, compound feeds and their bag residues after ruminal *in situ* incubation; cross-validation groups: 5

	Settings		Calibration				Validation				
Data set	Wavelength (nm)	D,G,S	Factors	Samples Available/used	SEC (µmol/g)	*R* ^2^	SEP (µmol/g)	*R* ^2^	Bias (µmol/g)	Slope	Intercept (µmol/g)
(1)	1250 to 2450	1,8,8	15	127/127	3.6	0.95	5.3	0.90	−0.76	1.04	−1.55
(2)	1250 to 2450	1,8,8	15	259/259	3.9	0.94	4.5	0.93	−0.43	1.02	−0.88
(3)	1250 to 2450	1,8,8	15	229/229	4.0	0.94	5.1	0.92	−0.61	1.03	−1.23
(4)	680 to 2500	1,8,8	5	95/87	1.5	0.92	4.2	0.66	<0.01	1.00	<0.01
(5)	1250 to 2450	1,8,8	15	156/156	3.2	0.97	4.6	0.95	−0.61	1.03	−1.24
(6)	1250 to 2450	1,8,8	15	220/220	3.7	0.95	4.2	0.94	−0.32	1.02	−0.64
(7)	1250 to 2450	1,8,8	15	117/117	3.3	0.97	3.9	0.97	−1.01	1.04	−2.06

D,G,S = Derivation, Gap, Smooth; *R*^2^ = squared correlation coefficient; SEC = Standard Error of Calibration; SEP = Standard Error of Prediction; data set 1: all values for feeds and bag residues of the present study; data set 2: all values for feeds and bag residues of the present study and the additional studies (Seifried *et al.*, 2016 and 2017; Haese *et al.*, 2017c, Krieg *et al.*, 2017); data set 3: data set 2, but excluding all values for rye and triticale; data set 4: only values for feeds and bag residues from grain samples of the present study and the additional studies; data set 5: data set 2, but excluding all values for grain samples; data set 6: data set 2, but excluding all values for compound feeds; data set 7: data set 2, but excluding all values for compound feeds and grain samples.

**Table 6:** Effective degradation of phytate (InsP_6_) at a passage rate of 5 (InsP_6_ED_5_) and 8 (InsP_6_ED_8_) %/h calculated from InsP_6_ concentrations predicted with near-infrared spectroscopy (NIRS) or chemically (HPIC) analysed

Feed	Maize		Wheat		Barley		Faba beans		Soybeans		Soybean meal		Rapeseed meal		Sunflower meal		DDGS		CF1 Mash		CF1 Pellet		CF2 Mash		CF2 Pellet			*P*-values		
Method	NIRS	HPIC	NIRS	HPIC	NIRS	HPIC	NIRS	HPIC	NIRS	HPIC	NIRS	HPIC	NIRS	HPIC	NIRS	HPIC	NIRS	HPIC	NIRS	HPIC	NIRS	HPIC	NIRS	HPIC	NIRS	HPIC	Pooled SEM	Feed × Method	Method	Feed
InsP_6_ED_5_	85^fg^	92^abc^	90^cde^	82^ h^	85^ g^	80^ h^	94^a^	94^ab^	88^ef^	89^de^	71^j^	76^i^	61^ k^	59^ k^	61^j^	75^i^	93^ab^	92^abc^	91^abcd^	86^fg^	92^abc^	91^bcde^	80^ h^	80^ h^	86^fg^	85^ g^	1.2	<0.001	0.643	<0.001
InsP_6_ED_8_	80^fg^	90^abc^	86^cde^	76^hi^	79^gh^	74^i^	92^a^	91^ab^	84^ef^	85^de^	59^ k^	66^j^	49^ l^	48^ l^	49^ l^	65^j^	92^a^	89^abc^	89^abc^	81^fg^	90^ab^	88^bcd^	73^i^	72^i^	81^fg^	80^ g^	1.0	<0.001	0.865	<0.001

DDGS = dried distillers’ grains with solubles; CF1 = compound feed 1 (containing 10% maize, 46% barley, 16% faba beans, 18% soybeans, 5% soybean meal, 5% DDGS on DM basis); CF2 = compound feed 2 (containing 32% maize, 12% wheat, 16% faba beans, 8% soybean meal, 17% rapeseed meal, 10% sunflower meal, 5% DDGS on DM basis).

Different superscripts within a row indicate significant differences.

## Discussion

### Phytate degradation from single feeds

The wide variation in InsP_6_ED between the examined feeds proves the necessity to evaluate the ruminal degradation of InsP_6_ individually for single feeds. The results showed that even when feeds are categorised in legume seeds (faba beans, soybeans), cereals (maize, wheat, barley) and oilseed meals (SFM, SBM, RSM), InsP_6_ED varies widely within these categories. For unprocessed feeds, the extent of ruminal InsP_6_ degradation seems to be influenced mainly by localisation and binding of InsP_6_ in the seeds (Haese *et al.*, 2017a and 2017b). However, the effects of genotype and harvest year on InsP_6_ degradation of legume seeds and cereal grains have not yet been studied. As variation of ruminal CP degradation between barley (ED_8_: 69% to 80%; Krieg *et al.*, 2018b) and wheat (ED_8_: 72% to 80%; Seifried *et al.*, 2017) genotypes has been observed, this might also apply to ruminal InsP_6_ degradation. In a previous study, we examined the correlation between CP and InsP_6_ disappearance for different feeds and found high coefficients of determination for the linear regressions (*R* ^2^ ≥ 0.93 for oilseed meals, *R* ^2^ = 0.83 for wheat; Haese *et al.*, 2017b). Therefore, factors influencing ruminal CP degradation might also affect ruminal InsP_6_ degradation.

For processed feeds such as oilseed meals, processing conditions seem to have a major influence on the extent of ruminal InsP_6_ degradation and might explain the relatively low InsP_6_ED of SBM and RSM compared to other studies. In the studies of Konishi *et al.* (1999) and Park *et al.* (1999), InsP_6_ED_8_ was 59% for RSM and 74% for SBM, while in the present study, InsP_6_ED_8_ was only 48% for RSM and 66% for SBM. Heat treatment seems to have a major influence on InsP_6_ED, as additional heating of meals for 3 h at different temperatures (133°C, 143°C, 153°C) reduced InsP_6_ED_8_ for both RSM (46%, 42%, 14%) and SBM (65%, 57%, 45%; Konishi *et al.* (1999)). Steingass *et al.* (2013) and Broderick *et al.* (2016) found considerable variation of ruminal degradability of CP in RSM from different oil mills and explained these observations with different heating procedures during toasting. Because disappearance of CP and InsP_6_ is correlated in oilseed meals (Haese *et al.*, 2017b), it is likely that ruminal InsP_6_ degradation in RSM and SBM also depends on the production process and thus differs between meals from different processing plants. The same might apply to SFM where, to the best of the authors’ knowledge, data on ruminal InsP_6_ degradation have not yet been published.

As no accumulation of InsP_3-5_ was observed for any incubated feed, it can be assumed that InsP_6_ is completely dephosphorylated once this process has begun on an InsP_6_ molecule. For poultry, it has been shown that, even when phytase is supplemented to the feed, InsP_6_ is not completely dephosphorylated in the precaecal part of the digestive tract (Sommerfeld *et al.*, 2018). In ruminants, however, the *in vitro* study of Brask-Pedersen *et al.* (2011) as well as the *in situ* study of Haese *et al.* (2017b) suggested that the crucial step in InsP_6_ degradation is the cleavage of the first phosphate group and hydrolysis of InsP_5_ and lower InsPs follows soon after. This is consistent with the results of the present study and can probably be assumed for all feedstuffs as a quite broad range of feeds was examined. Still little is known about phytase-producing bacteria and their specific phytases, but Nakashima *et al.* (2007) found two different phytase sequences in the rumen bacterium *Selenomonas lacticifex* and suggested that in this bacterium multiple phytate degrading enzymes are present. Furthermore, Li *et al.* (2014) found that phytase-producing microorganisms did not constantly secrete functional phytases, when rumen samples gained at different times after feeding were analysed. This indicates that in the rumen various phytases are available at any time leading to complete hydrolysis of InsP_6_, whereas in non-ruminants, where diets are usually supplemented with only one specific phytase, lower InsPs do accumulate.

### Additivity of phytate degradation of compound feeds and pelleting effect

Compound feeds are often pelleted, hence it is of practical value if InsP_6_ED can be calculated from that of single feeds. Calculated InsP_6_ED underestimated observed InsP_6_ED of both CF1 Pellet (InsP_6_ED_5_: 4, InsP_6_ED_8_: 6 percentage points) and CF2 Pellet (InsP_6_ED_5_: 8, InsP_6_ED_8_: 11 percentage points). This suggests that, at present, InsP_6_ED of CF cannot be calculated reliably with sufficient precision from values of single feeds. As the difference between calculated and observed values of InsP_6_ED was smaller for CF1, the precision of the calculation could depend on the single feeds used. So far, CF are mainly used to supply energy and CP, and their contribution to P supply has not yet been of major interest. However, depending on the constituent single feeds its contribution can be relevant, and gaining an estimate of the availability of this P source is an improvement towards precise calculation of diets. Thus, further research is required on this topic as we examined only two different CF in the present study.

Both CF1 Pellet and CF2 Pellet showed higher InsP_6_ED values compared to the respective Mash (CF1: InsP_6_ED_5_: 5, InsP_6_ED_8_: 7 percentage points; CF2: InsP_6_ED_5_: 5; InsP_6_ED_8_: 8 percentage points). This effect was also observed for effective degradation of CP in CF1 and CF2 (Grubješić *et al.*, 2019). As degradation rate *c* was not affected by pelleting, this effect can probably be ascribed to the increase of fraction *a* after pelleting (CF1: 15, CF2: 18 percentage points). A higher proportion of finer particles was measured after pelleting of CF1 and CF2 (Grubješić *et al.*, 2019), and it can be concluded that the increased InsP_6_ED in pelleted feeds derived from fine particles which were prone to leave the bag undegraded and thus increased fraction *a*. As mentioned before, heat treatment at high temperatures usually impairs ruminal InsP_6_ degradation. Pelleting proceeded at a temperature of 50°C to 60 °C, and the exit temperature of the pellets was 80°C to 90 °C. Either this temperature was not sufficient to facilitate any structural changes decreasing InsP_6_ degradation or the changes in particle size distribution covered this effect.

### Prediction of phytate concentrations using near-infrared spectroscopy

The performance of the calibration based on data set 7 yielded the highest *R* ^2^ in the validation step and the lowest SEP of all calibrations. Thus, the difference between the chemically analysed and NIRS predicted InsP_6_ concentrations were overall lower for data set 7 than for the other calibrations (Figure 2). However, the bias and intercept were higher for data set 7 calibrations than for the other sets. When regressions were calculated between the error of InsP_6_ predictions and the predicted InsP_6_ concentrations, slopes were not significant in any case. This implies that the error of the prediction did not depend on the InsP_6_ concentration of the sample. This, in turn, means that the prediction of InsP_6_ concentrations is possible with similar accuracy for feed samples and bag residues, where InsP_6_ concentrations are distinctly lower due to ruminal incubation.

Overall, the performance of calibrations in the present study was not as good as the performance of calibrations for the prediction of CP concentrations in similar samples (Krieg *et al.*, 2018a). For most of the data sets, the wavelength segment of 1250 to 2450 nm was selected for prediction of CP and InsP_6_ concentration. The aforementioned correlation between CP and InsP_6_ concentration in different feeds (Haese *et al.*, 2017b) and the preference for the same wavelength segments support the theory of InsP_6_ being indirectly predicted from CP. Since InsP_6_ and CP concentrations are correlated but do not change directly proportional, this theory would also explain the lower performance of InsP_6_ calibrations compared to the calibrations for predicting CP concentration.

The improvement of the performance of the calibrations by exclusion of cereal grains and CF suggests that strong matrix effects exist between cereal grain samples and protein feeds. No clear separation of spectra from cereal grain samples and their incubation residues from the other samples was visible (principal component analysis plot, data not shown, MATLAB, Fathom Toolbox; Jones (2014)). However, the decrease in the SEP and the increase in the *R* ^2^ upon exclusion of grain samples suggest that separate calibrations for cereal grains and protein-rich feeds should be further worked on. Assumedly, the matrix effects occur due to different interactions between InsP_6_ and CP in cereal grains and protein feeds which result in differing degradation kinetics of CP and InsP_6_. This probably leads to changes in the relations between InsP_6_ and CP concentrations of feeds and bag residues which might affect protein-rich feeds to a different extent than cereal grains. Together with the previously assumed indirect prediction of InsP_6_ by CP, this could lead to a less favourable performance of global calibrations. This theory is supported by the relatively homogenous distribution of the samples in the PCA plot. A separation of grain samples based on the error of the prediction could be expected based on the comparison of the InsP_6_ED values, but was not given for any of the calibrations (Figure 2). The comparison of InsP_6_ED NIRS with InsP_6_ED HPIC also indicates that the NIRS prediction of InsP_6_ concentrations is not yet sufficiently accurate. While no differences between InsP_6_ED NIRS and InsP_6_ED HPIC were observed for some feeds, InsP_6_ED NIRS was considerably lower (e.g. 16 percentage points for SFM) or higher (e.g. 10 percentage points for wheat) for other feeds. This underlines the need for more data to develop suitable calibrations.

The authors are not aware of any study that reported calibrations to predict InsP_6_ concentrations in ruminally incubated samples. However, calibrations do exist to predict InsP_6_-P concentration in poultry feeds (Tahir *et al.*, 2012; Aureli *et al.,* 2017). Values of the present study expressed as InsP_6_-P ranged from 0.23 to 12.12 g/kg, which is in a similar range as the values of Tahir *et al.* (2012) and Aureli *et al.* (2017). In the study of Tahir *et al.* (2012), the *R* ^2^ of the validation step ranged from 0.67 (maize) to 0.94 (wheat shorts) and the SEP from 0.09 g/kg (SBM) to 0.23 g/kg (maize, DDGS). Recalculation of the SEP in the present study to g/kg InsP_6_-P resulted in slightly higher SEP values between 0.7 and 1.0 g/kg. Calibrations of Aureli *et al.* (2017) were based on a slightly bigger range of reference InsP_6_-P concentrations (0.2 to 14.1 g/kg) and showed a comparable *R* ^2^ (0.94) and SEP (0.67 g/kg) than most of the calibrations of the present study. The slightly higher SEP values observed here are probably due to the more heterogeneous sample material (feeds and bag residues after different incubation times) compared to calibrations comprising only feedstuffs. Besides the establishment of local calibrations, the usage of other chemometric techniques than PLS might help to improve the accuracy of the prediction. First trials with data of the present study utilising artificial neural networks instead of PLS to predict InsP_6_ concentrations delivered promising results and should be further investigated. Overall, the calibrations that were established in the present study demonstrate that InsP_6_ can be predicted by NIRS in incubated samples of *in situ* studies as well as in feeds. However, the results also show that the used database needs to be expanded to achieve sufficient performance of the calibrations for the use in *in situ* studies.

The results of the present study indicate that the availability of InsP_6_-P should be evaluated individually for feeds. However, to broaden the data base on ruminal InsP_6_ degradation of different feeds establishing a fast and easy method for analysis of InsP_6_ is a decisive factor. Predicting InsP_6_ concentrations in feeds and bag residues using NIRS proved to have the potential to simplify the analytical step of InsP_6_ in future *in situ* studies.

## Software and data repository resources

None of the data were deposited in an official repository.
